# Laser cooling with intermediate state of spin–orbit coupling of LuF molecule

**DOI:** 10.1038/s41598-023-32439-1

**Published:** 2023-05-01

**Authors:** N. El-Kork, A. AlMasri Alwan, N. Abu El Kher, J. Assaf, T. Ayari, E. Alhseinat, M. Korek

**Affiliations:** 1 Department of Physics, Khalifa University, P.O. Box 57, Abu Dhabi, United Arab Emirates; 2 Space and Planetary Science Centre, Khalifa University, Abu Dhabi, United Arab Emirates; 3 Doctoral School of Sciences and Technology, Lebanese University, Hadath, Lebanon; 4 Center for Educational Research and Development, CERD, Sin El Fil, Lebanon; 5 Faculty of Science, Beirut Arab University, Riad El Solh, Beirut, 1107 2809 Lebanon

**Keywords:** Atomic and molecular physics, Chemical physics

## Abstract

This work presents a theoretical study of the laser cooling feasibility of the molecule LuF, in the fine structure level of approximation. An ab-initio complete active space self-consistent field (CASSCF)/MRCI with Davidson correction calculation has been done in the Λ^(±)^ and Ω^(±)^ representations. The corresponding adiabatic potential energy curves and spectroscopic parameters have been investigated for the low-lying electronic states. The calculated values of the internuclear distances of the X^3^Σ_0+_ and (1)^3^Π_0+_ states show the candidacy of the molecule LuF for direct laser cooling. Since the existence of the intermediate (1)^3^Δ_1_ state cannot be ignored, the investigation has been done by taking into consideration the two transitions (1)^3^Π_0+_−(1)^3^Δ_1_ and (1)^3^Π_0+_ −X^3^Σ_0+_. The calculation of the Franck–Condon factors, the radiative lifetimes, the total branching ratio, the slowing distance, and the laser cooling scheme study prove that the molecule LuF is a good candidate for Doppler laser cooling.

## Introduction

Cold and ultracold molecules offer new insights into many-body physics, revolutionize physical chemistry, and provide techniques for probing new states of quantum matter. They represent an exciting new frontier that enables the investigation of measurements at an unprecedented level of detail. Besides, polar ultracold molecules present new platforms for quantum information, quantum computing^1–4^, and quantum simulation of many-body interactions^5,6^. Dipole–dipole interactions among ultracold polar molecules, such as LuF can lead to discoveries beyond traditional molecular science^7^.

The group of lutetium mono-halides LuX (X = F, Cl, Br, I) has been the subject of numerous experimental and theoretical studies and has an increasing interest in various types of research^8–11^. LuF is an element of the lutetium mono-halides LuX group, which is significant in astrophysics due to their presence in many stars enriched by the r-nucleosynthesis process^12–15^, in the interstellar medium^16^, and in the cool stellar atmospheres^17^. This molecule has been studied experimentally in literature^18–21^, while the theoretical studies in Λ^(±)^ representation are given in Refs.^22–28^. The Ω^(±)^ states of LuF molecules have been investigated by Assaf et al.^29^, where 36 electronic states have been investigated.

## Computation and results

**Figure 1:**
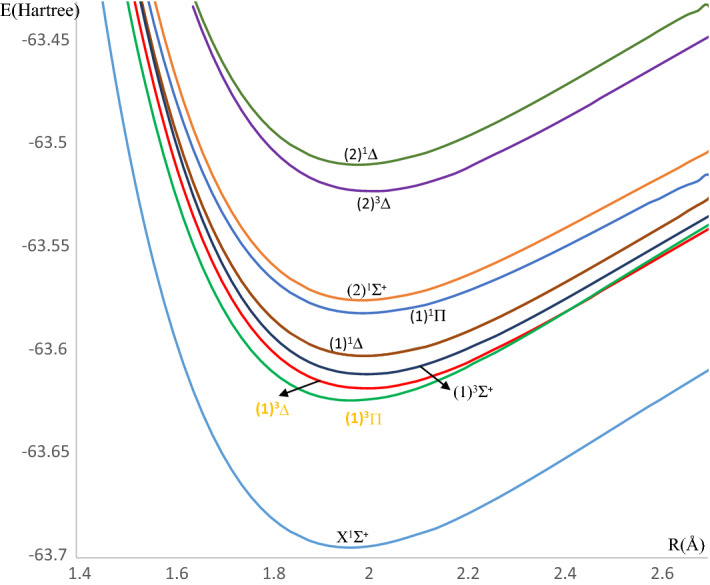
The potential energy curves of the low-lying free spin singlet and triplet electronic states of LuF molecule.

We initially performed preliminary investigations of the electronic structure of the LuF molecule in the spin-free approximation. The Quantum Chemistry Package Molpro 2010^30^ was used by applying the *ab-initio* Complete Active Space Self-consistent field (CASSCF) method. The adiabatic potential energy curves have been calculated by employing the internally contracted Multi Reference Configuration interaction plus Davidson correction (MRCI + Q) techniques within the Born–Oppenheimer approximation. In the Λ^(±)^ representation, the adopted basis sets for the Lu and F atoms are respectively ECP60MWB (using *s*, *p*, *d* atomic orbitals) and ECP2SDF (using *s*, *p* atomic orbitals). The investigated potential energy curves in this representation for the lower electronic states are shown in Fig. 1. We can note that the first low-lying states of the LuF molecule are of triplet multiplicity as in all electronic structures reported previously^27–29^. The spectroscopic constants T_e_, R_e_, ω_e_, ${\omega }_{e}{x}_{e},$ B_e_, α_e_, and *D*_*e*_ have been calculated by fitting the potential energy curve values around the minimum of the internuclear distance R_e_ to a polynomial in terms of R, the significant degree of which is determined from the evaluation of the statistical error for the coefficients, for the ground and the low-lying states of the LuF molecule, as presented in Table 1. In this Table, the comparison of our calculated value of R_e_ for the ground and the investigated excited electronic states with those given in the literature proved a suitable accuracy with relative difference values that are less than 3%.

**Table 1:** The spectroscopic constants of the spin-free ^2s+1^Ʌ^(+/−)^ electronic states of LuF molecule.

States	Ref.	R_e_ (Å)	$\Delta {R}_{e}/{R}_{e}$ %	T_e_ (cm^−1^)	$\Delta {T}_{e}/{T}_{e}$ %	$\upomega$_e_ (cm^−1^)	$\Delta {\upomega }_{e}/{\omega }_{e}$ %	${\omega }_{e}{x}_{e}$ (cm^−1^)	$\Delta {\upomega }_{e}{x}_{e}/{\omega }_{e}{x}_{e}$ %	B_e_ (cm^−1^)	$\Delta {B}_{e}/{B}_{e}$ %	${\alpha }_{e}$×10^3^ (cm^−1^)	${D}_{e}$×10^6^ (cm^−1^)
X^1^Σ^+^	Present work	1.962		0		614.15		2.60		0.256		1.55	0.181
	a	1.922	*2.0*	0	*0.0*	606.6	*1.2*	3.30	*21.2*	−		−	−
	b	1.918	*2.2*	0	*0.0*	612.91	*0.2*	2.52	*3.1*	−		−	−
	c	1.913	*2.5*	0	*0.00*	618.89	*0.8*	2.53	*2.7*	−		−	−
	**d**	−		−		**610.6**	***0.6***	**2.5**	***3.8***	−		−	−
	**e**	**1.9165**	***2.3***	**0**	***0.0***	**611.79**	***0.4***	**2.54**	***2.3***	**0.268**	***4.5***	**1.56**	**0.205**
	**f**	**1.917**	***2.3***	**−**		**618**	***0.6***	**2.82**	***7.8***	**−**		**−**	**−**
(1)^3^Π	Present work	1.967		15 684		576.95		2.33		0.254		1.685	0.197
	a	1.923	*2.2*	16 165	*3.0*	596.2	*3.2*	3.0	*22.3*	−		−	−
	b	1.928	*2.0*	16 602	*5.5*	590.96	*2.4*	2.66	*12.4*	−		−	−
	c	1.933	*1.7*	15 956	*1.7*	579.22	*0.4*	2.67	*12.7*	−		−	−
(1)^3^Δ	Present work	1.997		16 962		551.68		3.0		0.247		0.815	0.207
	a	1.952	*2.3*	17 904	*5.3*	561.00	*1.7*	3.40	*11.8*	−		−	−
	b	1.946	*2.6*	16 653	*1.8*	572.9	*3.7*	3.52	*14.8*	−		−	−
	c	1.947	*2.5*	14 927	*12.0*	576.04	*4.2*	2.71	*9.7*	−		−	−
(1)^3^Σ^+^	Present work	1.997		18 484		548.33		2.73		0.247			
	a	1.953	2.2	19 131	*3.4*	567.1	*3.3*	2.6	*4.8*	−		−	−
	b	1.955	2.1	19 296	*4.2*	570.22	*3.8*	2.6	*4.8*	−		−	−
	c	1.961	1.8	18 856	*2.0*	559.57	*2.0*	2.50	*8.4*	−		−	−
(1)^1^Δ	Present work	1.992		20 428		567.43		2.55		0.248		2.24	0.189
	a	1.956	*1.8*	21 634	*5.6*	555.0	*2.2*	2.5	*2.0*	−		−	−
	b	1.952	*2.0*	21 137	*3.4*	568.99	*0.3*	2.57	*0.8*	−		−	−
	c	1.955	*1.9*	19 471	*4.7*	567.71	*0.0*	2.81	*9.3*	−		−	−
(1)^1^Π	Present work	1.988		24 973		544.28		2.57		0.249		2.35	0.221
	a	1.945	*2.2*	25 538	*2.2*	544.7	*0.1*	2.6	*1.2*	−		−	−
	b	1.958	*1.5*	24 633	*1.4*	540.48	*0.7*	2.22	*13.6*	−		−	−
	c	1.972	*0.8*	23 708	*5.1*	525.34	*3.5*	2.18	*15.2*	−		−	−
	**d**	−		−		**542.6**	***0.3***	**2.3**	***10.5***	−		−	−
	**e**	**1.958**	***1.5***	**24 465.68**	***2.0***	**543.42**	***0.2***	**2.28**	***11.3***	**0.256**	***2.7***	**1.61**	**0.228**

Percentage relative error values are in [italics].

^a^Ref. ^27^, ^b^Ref. ^28^, ^c^Ref. ^29^, ^d^Ref. ^18^, ^e^Ref. ^19,20^, ^f^Ref. ^21^. Experimental values are indicated in bold.

Similarly, the values of T_e_ and ω_e_ also present a good agreement for the six studied states with relative differences close to 12% compared with the literature. Comparing our values with the experimental data^18–21^ for the ground state proves a high accuracy with average percentage errors of 4.6%, 4.5%, 0.6%, and 11.7% for ${\omega }_{e}{x}_{e, }$ B_e_, α_e_, and *D*_*e*_ respectively. Furthermore, the electronic spectrum of the LuF molecule has been recorded experimentally using a hollow cathode lamp by D’Incan et al.^18^ and Effantin et al.^19^; they assigned the following notation for the excited states : A^1^Σ^+^, B^1^Π, D^1^Π, E^1^Π, and F^1^Σ^+^. However, similarly to Assaf et al.^29^ and Hamadeh et al.^27^, in our work, the first excited state was found to be a ^1^Π state, with a transition energy T_e_ = 24 973 cm^−1^. This state doesn’t correspond to the observed A, B, or D systems, but to the reported E^1^Π state, where the spectroscopic constants average relative differences with our calculations are 2.0% for Te, 1.5% for Re, 0.3% for ω_e_ and 2.7% for Be.

**Figure 2:**
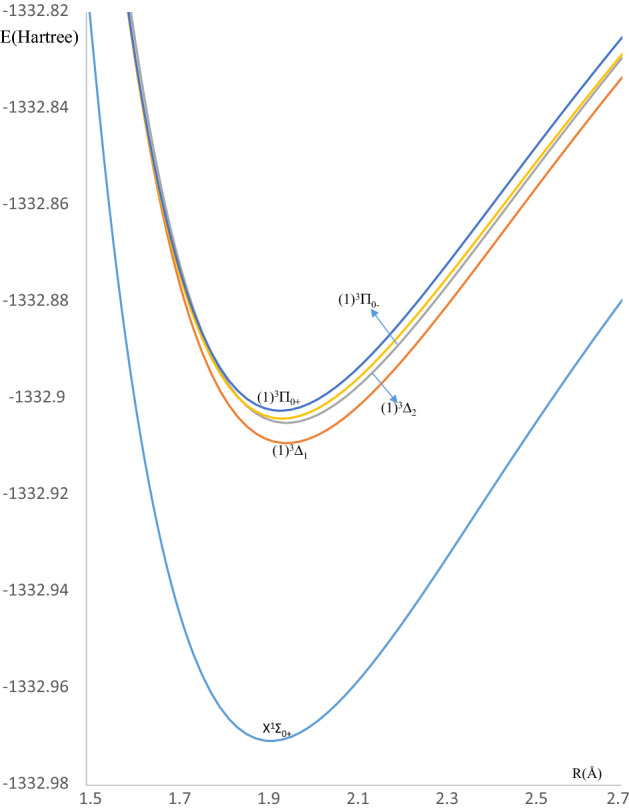
The potential energy curves of the low-lying spin orbit states of LuF molecule.

**Figure 3:**
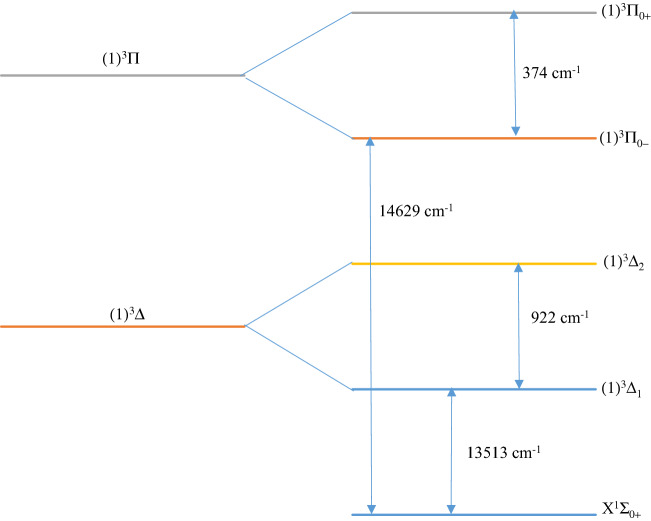
Values of the splitting energy of ^3^Π and ^3^Δ of the molecule LuF.

We perform our calculations with Spin–Orbit Coupling (S.O.C) effects for the LuF molecule in the Ω^(±)^ representation for a more accurate description of experimentally observed systems. We use the same basis sets ECP28-MWB with ANO-SO^31^ for the Lu atom and an all-electrons scheme for the F atom^32^ as presented by Assaf et al.^29^. The potential energy curves and the splitting energies for the three lowest electronic states X^1^Σ^+^ (X^1^Σ_0+_), (1)^3^Π ((1)^3^Π_0+_, ^3^Π_0−_), and (1)^3^Δ ((1)^3^Δ_**1**_, (1)^3^Δ_**2**_) are respectively given in Figs. 2 and 3. Also, the accurate spectroscopic constants of the bound Ω states of LuF molecule have been calculated and listed in Table 2 with the same method used for the spin-free states. As shown in Figs. 2 and 3, we find relatively large values of the splitting energies for ^3^Π (about 375 cm^−1^) and ^3^Δ states (about 930 cm^−1^) indicating the significant effects of the spin–orbit coupling on the electronic states of the LuF molecule. Effantin et al. observed five bands for singlet transition systems: A^1^Σ^+^$\to$ X^1^Σ^+^, B^1^Π $\to$ X^1^Σ^+^, D^1^Π $\to$ X^1^Σ^+^, E^1^Π $\to$ X^1^Σ^+^, and F^1^Σ^+^$\to$ X^1^Σ^+^. As discussed by Hamade et al.^27^ a comparison of the observed levels with those obtained throught our calculations, shows that the upper states A and B are not equivalent to any ^1^Σ^+^ and ^1^Π. Rather, the transition A^1^Σ^+^$\to$ X^1^Σ^+^ predicted by Effantin et al. shows a band of $\Delta\Omega =0$, which is attributed to the spin–orbit transition ^3^Π_0+_$\to$ X^1^Σ_0+_. On other hand, the observed transition B^1^Σ^+^$\to$ X^1^Σ^+^ transition of $\Delta\Omega =1$, is equivalent to that of ^3^Π_1_ $\to$ X^1^Σ_0+_. These results led us to confirm that the upper states A^1^Σ^+^ and B^1^Π are the components ^3^Π_0+_ and ^3^Π_1_ of (1)^3^Π state respectively. Consequently, our calculations show a good agreement with the experiment conducted by Effantin et al. for the (1)^3^Π_0+_ state with a relative discrepancy $\Delta {R}_{e}/{R}_{e}=0.2 \%$, $\Delta {T}_{e}/{T}_{e}=7.2 \%$, $\Delta {\upomega }_{e}/{\omega }_{e}=0.5 \%$, $\Delta {{\omega }_{e}x}_{e}/{{\omega }_{e}x}_{e}=4.6 \%$, and $\Delta {\mathrm{B}}_{e}/{B}_{e}=0.4 \%$.

**Table 2:** The spectroscopic constants of the spin–orbit ${\Omega }^{(\pm )}$ electronic states of LuF molecule.

States	Ref.	R_e_ (Å)	$\Delta {R}_{e}/{R}_{e}$ %	T_e_ (cm^−1^)	$\Delta {T}_{e}/{T}_{e}$ %	$\upomega$_e_ (cm^−1^)	$\Delta {\upomega }_{e}/{\omega }_{e}$ %	${\omega }_{e}{x}_{e}$ (cm^−1^)	$\Delta {\upomega }_{e}{x}_{e}/{\omega }_{e}{x}_{e}$ %	B_e_ (cm^−1^)	$\Delta {B}_{e}/{B}_{e}$ %	${\alpha }_{e}$×10^3^ (cm^−1^)	${D}_{e}$×10^6^ (cm^−1^)
X^1^Σ_0+_	Present work	1.914		0		618.93		2.77		0.268		1.62	0.202
	a	1.914	*0.0*	0	*0.0*	619.36	*0.1*	2.53	*8.7*	−		−	−
	**b**	−		−		**610.6**	***1.3***	**2.5**	***9.7***	−		−	−
	**c**	**1.9165**	***0.13***	**0**	***0.0***	**611.79**	***1.2***	**2.54**	***8.3***	**0.268**	***0.0***	**1.56**	**0.205**
(1)^3^Δ_1_	Present work	1.949		13 534.68		572.75		2.54		0.259		1.55	0.212
	a	1.949	*0.0*	13 513	*0.16*	572.44	*0.1*	2.57	*1.2*	−		−	−
(1)^3^Δ_2_	Present work	1.949		14,453.39		572.29		2.56		0.259		1.55	0.212
	a	1.949	*0.0*	14 435	*0.1*	572.74	*0.1*	2.82	*9.2*	−		−	−
(1)^3^Π_0−_	Present work	1.939		14 645.20		571.81		2.86		0.261		1.61	0.219
	a	1.938	*0.1*	14 629	*0.1*	573.32	*0.3*	2.58	9.8	−		−	−
(1)^3^Π_0+_	Present work	1.935		15 007.58		585.13		2.47		0.263		1.59	0.218
	a	1.935	*0.0*	15 003	*0.0*	577.29	*1.3*	2.69	*8.2*	−		−	−
	**b**	−		−		**586.4**	***0.2***	**2.5**	***1.2***	−		−	−
	**c**	**1.9313**	***0.2***	**16 164.64**	***7.2***	**587.95**	***0.5***	**2.59**	***4.6***	**0.264**	***0.4***	**1.62**	**0.208**

Percentage relative error values are in [italics].

^a^Ref. ^29^, ^b^Ref. ^18^, ^c^Ref. ^19,20^. Experimental values are indicated in bold.

We also performed a rovibrational study of the investigated states using the canonical function approach^33–35^ and cubic spline interpolation between every two consecutive points of the potential energy curves. Table 3 shows the vibrational energy Ev, the rotational constant ${B}_{v}$, and the centrifugal distortion constant ${D}_{v}$ for the investigated spin-free (Λ^(±)^ representation) and spin–orbit (Ω^(±)^ representation) curves, with a comparison with previously published data. Our values for the different vibrational levels of the (X)^1^Σ^+^, (1)^1^Π, X^1^Σ_0+_, and (1)^3^Π_0+_ states of LuF molecule agree well with the experimental ones^19,21,22^, with an relative difference of $0.3 \%\le \Delta {E}_{v}/{E}_{v}\le 2.6 \%$, $0.3 \%\le \Delta {B}_{v}/{B}_{v}\le 5.1 \%$, and $0 \%\le \Delta {\mathrm{D}}_{v}/{D}_{v}\le 21.5 \%$. We compared in Table 3 the ro-vibrational constants values for X^1^Σ^+^ that were reported by Effantin et al.^19^ with our calculated X^1^Σ^+^ and X^1^Σ_0+_ values to confirm that this state is of X^1^Σ_0+_ nature. In fact, the values of ${B}_{v}$ and ${D}_{v}$ of X^1^Σ_0+_ are closer to experimental data than those for X^1^Σ^+^, as previously discussed. Generally, our present calculations agree with the available experimental values, which confirms the credibility of our work. In addition, Table 4 shows the ro-vibrational constant values for the remaining low-lying excited states of the LuF molecule. No comparison has been reported for these levels since they are given here for the first time.

**Table 3:** Values of the eigenvalues, and the rotational constants for the different vibrational levels of the (X)^1^Σ^+^, (1)^1^Π, X^1^Σ_0+_, and (1)^3^Π_0+_ states of LuF molecule comparison with the experimental values.

State	$v$	${E}_{v}$ (cm^−1^)	$\Delta {E}_{v}/{E}_{v}$ %	${B}_{v}$ (cm^−1^)	$\Delta {B}_{v}/{B}_{v}$ %	${D}_{v}$×10^7^ (cm^−1^)	$\Delta {D}_{v}/{D}_{v}$ %
(X)^1^Σ^+^	0	306.09 − − 305.24^c^	***0.3***	0.2544 **0.2669**^**a**^ **0.2669**^**b**^ **−**	***4.7*** ***4.7***	1.82 **2.02**^**a**^ − **−**	***9.9***
	1	905.94 − − 911.91^c^	***0.7***	0.2529 **0.2653**^**a**^ **0.2653**^**b**^ **−**	***4.7*** ***4.7***	1.85 **2.03**^**a**^ − **−**	***8.9***
	2	1497.36 1513.50^c^	***1.1***	0.2509 **0.2638**^**a**^ **0.2637**^**b**^ **−**	***4.9*** ***4.9***	2.02 **2.07**^**a**^ − **−**	***2.4***
	3	2072.44 2110.01^c^	***1.8***	0.2489 **0.2622**^**a**^ **−**	***5.1***	2.13 **2.03**^**a**^ **−**	***4.7***
	4	2634.09 − 2701.45^c^	***2.5***	0.2479 **0.2606**^**a**^ **−**	***4.9***	1.70 **2.15**^**a**^ **−**	***20.9***
	5	3201.05 3287.80^c^	***2.6***	0.2474 **−**		1.52 **−**	
	6	3774.33 3869.08^c^	***2.4***	0.2458 **−**		1.79 **−**	
	7	4343.23 4445.28^c^	***2.3***	0.2441 **−**		1.85 **−**	
	8	4908.06 5016.39^c^	***2.2***	0.2433 **−**		1.94 **−**	
	9	5468.05 5582.43^c^	***2.0***	0.2422 **−**		1.81 **−**	
	10	6024.00 6143.39^c^	***1.9***	0.2405 **−**		1.55 **−**	
	11	6579.43 6699.26^c^	***1.8***	0.2393 **−**		1.89 **−**	
	12	7129.85 7250.06^c^	***1.7***	0.2381 **−**		1.90 **−**	
	13	7675.59 7795.77^c^	***1.5***	0.2369 **−**		1.74 **−**	
	14	8218.25 8336.40^c^	***1.4***	0.2355 **-**		1.72 **-**	
	15	8757.58		0.2342		1.73	
	16	9293.71		0.2330		1.90	
	17	9825.33		0.2318		1.80	
	18	10,353.34		0.2306		1.75	
	19	10,877.89		0.2293		1.78	
	20	11,398.89		0.2281		1.85	
	21	11,915.89		0.2270		1.76	
	22	12,429.47		0.2257		1.75	
	23	12,939.64		0.2245		1.77	
	24	13,446.27		0.2233		1.75	
	25	13,949.48		0.2221		1.78	
	26	14,449.10		0.2209		1.80	
	27	14,944.97		0.2197		1.84	
	28	15,436.68		0.2183		1.88	
	29	15,923.88		0.2170		1.90	
	30	16,406.59		0.2157		1.77	
	31	16,885.95		0.2147		1.53	
	32	17,363.70		0.2138		1.55	
	33	17,839.58		0.2127		1.85	
	34	18,311.45		0.2114		1.88	
	35	18,779.22		0.2103		1.45	
	36	19,245.77		0.2097		1.35	
	37	19,711.75		0.2088		1.63	
	38	20,175.42		0.2079		1.54	
	39	20,637.04		0.2070		1.65	
	40	21,095.66		0.2056		2.23	
	41	21,547.86		0.2038		2.03	
	42	21,994.96		0.2028		1.44	
	43	22,440.43		0.2020		1.48	
	44	22,884.18		0.2010		1.67	
	45	23,325.08		0.1999		1.82	
	46	23,762.34		0.1988		1.54	
	47	24,197.39		0.1980		1.45	
	48	24,630.69		0.1970		1.70	
	49	25,061.06		0.4071		39.34	
	50	25,489.11		0.4057		53.98	
	51	25,915.13		0.4037		74.36	
	52	26,339.00		0.4009		101.72	
	53	26,760.48		0.3883		98.08	
	54	27,180.01		0.3850		104.46	
	55	27,597.29		0.3874		401.55	
	56	28,011.78		0.4311		14.87	
	57	28,423.21		0.4305		23.68	
	58	28,829.96		0.4298		34.53	
	59	29,231.13		0.4288		48.13	
	60	29,626.79		0.4276		58.21	
	61	30,018.94		0.4254		93.97	
	62	30,409.89		0.4232		114.06	
	63	30,798.70		0.4203		134.71	
	64	31,183.37		0.4165		152.06	
	65	31,564.11		0.4117		161.84	
	66	31,943.20		0.4042		211.34	
	67	32,319.77		0.4432		3.09	
	68	32,692.66		0.4430		4.75	
	69	33,063.28		0.4426		4.24	
	70	33,431.98		0.4422		2.18	
	71	33,797.67		0.4416		2.90	

State	$v$	${E}_{v}$ (cm−^1^)	$\Delta {E}_{v}/{E}_{v}$ %	${B}_{v}$ (cm^−1^)	$\Delta {B}_{v}/{B}_{v}$ %	${D}_{v}$×10^7^ (cm^−1^)	$\Delta {D}_{v}/{D}_{v}$ %
(1)^1^Π	0	267.12		0.2474 **0.2555**^**a**^	***3.2***	2.17 **2.28**^**a**^	***4.8***
	1	791.88		0.2448 **0.2539**^**a**^	***3.6***	2.29 **2.27**^**a**^	***0.9***
	2	1302.89		0.2431 **0.2523**^**a**^	***3.6***	1.83 **2.26**^**a**^	***19.0***
	3	1826.50		0.2424 **0.2507**^**a**^	***3.3***	1.92 **2.4**^**a**^	***20***
	4	2352.27		0.2408 **0.2493**^**a**^	***3.4***	2.16 **2.75**^**a**^	***21.5***
	5	2870.89		0.2395		2.07	
	6	3386.00		0.2383		1.98	
	7	3898.69		0.2368		1.97	
	8	4407.96		0.2352		1.80	
	9	4916.42		0.2338		2.15	
	10	5419.23		0.2326		1.99	
	11	5919.03		0.2313		2.09	
	12	6414.17		0.2299		1.72	
	13	6908.05		0.2284		1.99	
	14	7397.98		0.2272		1.97	
	15	7884.54		0.2259		1.99	
	16	8367.51		0.2247		1.92	
	17	8847.21		0.2233		1.79	
	18	9324.29		0.2220		1.92	
	19	9798.02		0.2207		1.94	
	20	10,268.45		0.2195		1.94	
	21	10,735.52		0.2183		1.91	
	22	11,199.16		0.2169		2.01	
	23	11,658.60		0.2155		2.17	
	24	12,112.52		0.2138		2.36	
	25	12,559.51		0.2120		2.33	

State	$v$	${E}_{v}$ (cm^−1^)	$\Delta {E}_{v}/{E}_{v}$ %	${B}_{v}$ (cm^−1^)	$\Delta {B}_{v}/{B}_{v}$ %	${D}_{v}$×10^7^ (cm^−1^)	$\Delta {D}_{v}/{D}_{v}$ %
X^1^Σ_0+_	0	308.99		0.2678 **0.2669**^**a**^	***0.3***	2.03 **2.02**^**a**^	***0.5***
	1	922.72		0.2663 **0.2653**^**a**^	***0.4***	2.03 **2.03**^**a**^	***0.0***
	2	1531.26		0.2648 **0.2638**^**a**^	***0.4***	2.03 **2.07**^**a**^	***1.9***
	3	2134.66		0.2633 **0.2622**^**a**^	***0.4***	2.04 **2.03**^**a**^	***0.5***
	4	2733.03		0.2618 **0.2606**^**a**^	***0.5***	2.04 **2.15**^**a**^	***5.1***
	5	3326.41		0.2603		2.04	
	6	3914.88		0.2587		2.04	
	7	4498.49		0.2573		2.04	
	8	5077.31		0.2558		2.04	
	9	5651.39		0.2543		2.04	
	10	6220.78		0.2528		2.04	
	11	6785.55		0.2513		2.04	
	12	7345.73		0.2499		2.04	
	13	7901.35		0.2484		2.04	
	14	8452.41		0.2469		2.05	
	15	8998.87		0.2455		2.05	
	16	9540.74		0.2440		2.06	

State	$v$	${E}_{v}$ (cm^−1^)	$\Delta {E}_{v}/{E}_{v}$ %	${B}_{v}$ (cm^−1^)	$\Delta {B}_{v}/{B}_{v}$ %	${D}_{v}$×10^7^ (cm^−1^)	$\Delta {D}_{v}/{D}_{v}$ %
(1)^3^Π_0+_	0	289.92		0.2619 **0.2628**^**a**^	***0.3***	2.18 **2.07**^**a**^	***5.0***
	1	861.60		0.2603 **0.2611**^**a**^	***0.3***	2.19 **2.07**^**a**^	***5.5***
	2	1428.10		0.2587 **0.2596**^**a**^	***0.3***	2.19 **2.15**^**a**^	***1.8***
	3	1989.46		0.2571 **0.2579**^**a**^	***0.3***	2.19 **2.04**^**a**^	***6.8***
	4	2545.93		0.2555 **0.2563**^**a**^	***0.3***	2.20 **2.16**^**a**^	***1.8***
	5	3097.26		0.2539		2.19	
	6	3643.86		0.2523		2.20	
	7	4185.46		0.2508		2.20	
	8	4722.21		0.2492		2.19	
	9	5254.27		0.2476		2.22	
	10	5781.36		0.2460		2.19	
	11	6303.83		0.2445		2.20	
	12	6821.58		0.2429		2.22	
	13	7334.51		0.2414		2.19	
	14	7842.94		0.2398		2.21	
	15	8346.73		0.2383		2.22	
	16	8845.88		0.2367		2.19	

Percentage relative error values are in [italics].

^a^Ref.^19^, ^b^Ref.^21^, ^c^Ref.^22^. Experimental values are indicated in bold.

**Table 4:** Values of the eigenvalues, and the rotational constants for the different vibrational levels of the low-lying states (1)^1^Δ, (2)^1^Σ^+^, (2)^1^Δ, (1)^3^Δ, (1)^3^Σ^+^, (2)^3^Δ, and (1)^3^Π of LuF molecule.

State	$v$	${E}_{v}$ (cm^−1^)	${B}_{v}$ (cm−^1^)	${D}_{v}$×10^7^ (cm^−1^)
(1)^1^Δ	0	285.45	0.2470	1.91
	1	844.51	0.2451	2.07
	2	1384.91	0.2428	1.95
	3	1922.40	0.2421	1.44
	4	2478.01	0.2416	2.05
	5	3023.39	0.2396	2.10
	6	3560.15	0.2393	1.62
	7	4100.21	0.2376	2.06
	8	4631.67	0.2361	1.30
	9	5168.67	0.2348	2.24
	10	5695.63	0.2337	1.70
	11	6221.90	0.2327	2.17
	12	6740.65	0.2313	1.39
	13	7260.54	0.2299	1.95
	14	7775.19	0.2287	1.73
	15	8287.59	0.2276	2.07
	16	8794.49	0.2265	1.64
	17	9299.35	0.2250	1.75
	18	9800.97	0.2239	1.77
	19	10,299.37	0.2226	1.80
	20	10,794.44	0.2215	1.89
	21	11,285.70	0.2204	1.73
	22	11,774.06	0.2192	1.81
	23	12,259.04	0.2180	1.81
	24	12,740.76	0.2169	1.81
	25	13,219.23	0.2158	1.78
	26	13,694.62	0.2147	1.70
	27	14,167.30	0.2136	1.69
	28	14,637.38	0.2126	1.64
	29	15,105.25	0.2116	1.57
	30	15,571.48	0.2108	1.54
	31	16,036.29	0.2099	1.63
	32	16,498.82	0.2088	1.90
	33	16,957.05	0.2074	2.01
	34	17,410.40	0.2062	1.58
	35	17,861.88	0.2056	1.16
	36	18,314.20	0.2051	1.36
	37	18,766.02	0.2042	1.56
	38	19,216.01	0.2035	1.34
	39	19,665.26	0.2027	1.69
	40	20,111.24	0.2011	2.67
	41	20,548.26	0.1989	2.44
	42	20,977.96	0.1977	1.33
	43	21,406.64	0.1971	1.37
	44	21,834.37	0.1962	1.75
	45	22,258.90	0.1950	1.95
	46	22,679.15	0.1937	1.66
	47	23,096.78	0.1930	1.33
	48	23,513.27	0.1920	1.87

State	$v$	${E}_{v}$ (cm^−1^)	${B}_{v}$ (cm^−1^)	${D}_{v}$×10^7^ (cm^−1^)
(2)^1^Σ^+^	0	281.85	0.2481	1.97
	1	835.56	0.2459	2.04
	2	1377.97	0.2442	1.77
	3	1927.14	0.2433	1.82
	4	2476.49	0.2419	2.01
	5	3018.93	0.2407	2.06
	6	3554.49	0.2395	1.85
	7	4087.64	0.2380	1.78
	8	4618.12	0.2361	1.71
	9	5147.01	0.2349	2.09
	10	5669.17	0.2336	2.01
	11	6186.57	0.2325	1.95
	12	6699.22	0.2308	1.61
	13	7210.38	0.2293	1.95
	14	7716.67	0.2281	1.94
	15	8218.83	0.2268	2.03
	16	8716.05	0.2255	1.78
	17	9210.01	0.2240	1.78
	18	9700.53	0.2227	1.90
	19	10,186.99	0.2214	1.93
	20	10,669.40	0.2201	1.91
	21	11,147.82	0.2188	1.85
	22	11,622.57	0.2175	1.87
	23	12,093.53	0.2162	1.91
	24	12,560.51	0.2149	1.94
	25	13,023.37	0.2136	1.92
	26	13,482.17	0.2122	1.84
	27	13,937.47	0.2110	1.72
	28	14,390.32	0.2100	1.62
	29	14,841.44	0.2090	1.68
	30	15,290.27	0.2079	1.87
	31	15,735.36	0.2065	1.99
	32	16,175.88	0.2051	1.95
	33	16,612.25	0.2039	1.73
	34	17,045.98	0.2030	1.54
	35	17,478.18	0.2020	1.80
	36	17,907.24	0.2007	1.95
	37	18,332.36	0.1996	1.54
	38	18,755.62	0.1986	1.88
	39	19,175.00	0.1970	2.37
	40	19,587.97	0.1954	1.94
	41	19,996.70	0.1941	1.95
	42	20,401.55	0.1929	1.63
	43	21,204.71	0.1908	2.16
	44	21,600.52	0.1895	1.69
	45	21,993.64	0.1885	1.64
	46	22,384.51	0.1875	1.72

State	$v$	${E}_{v}$ (cm^−1^)	${B}_{v}$ (cm^−1^)	${D}_{v}$×10^7^ (cm^−1^)
**(2)**^**1**^**Δ**	0	298.32	0.2500	1.88
	1	872.50	0.2483	1.90
	2	1438.61	0.2462	1.96
	3	1996.00	0.2451	1.62
	4	2561.73	0.2443	1.98
	5	3120.09	0.2431	2.05
	6	3669.88	0.2417	1.55
	7	4222.67	0.2402	1.86
	8	4770.06	0.2386	1.67
	9	5316.33	0.2375	2.15
	10	5854.51	0.2365	1.87
	11	6388.61	0.2349	1.54
	12	6921.84	0.2334	1.89
	13	7450.22	0.2323	1.84
	14	7974.84	0.2310	1.90
	15	8495.10	0.2299	1.88
	16	9011.11	0.2285	1.62
	17	9524.69	0.2270	1.81
	18	10,034.58	0.2259	2.00
	19	10,539.62	0.2248	1.72
	20	11,041.60	0.2234	1.75
	21	11,540.25	0.2222	1.84
	22	12,034.65	0.2210	1.72
	23	12,525.92	0.2197	1.75
	24	13,013.98	0.2187	1.83

State	$v$	${E}_{v}$ (cm^−1^)	${B}_{v}$ (cm^−1^)	${D}_{v}$×10^7^ (cm^−1^)
**(1)**^**3**^**Δ**	0	278.67	0.2464	1.95
	1	832.20	0.2459	1.81
	2	1392.89	0.2445	1.91
	3	1948.86	0.2437	1.74
	4	2506.56	0.2420	1.92
	5	3057.91	0.2411	1.54
	6	3615.45	0.2406	1.90
	7	4167.59	0.2390	1.78
	8	4715.63	0.2374	2.04
	9	5255.11	0.2359	2.00
	10	5787.96	0.2347	1.59
	11	6320.48	0.2333	2.04
	12	6846.37	0.2321	1.75
	13	7369.55	0.2308	1.87
	14	7888.41	0.2295	1.83
	15	8403.70	0.2284	1.79
	16	8915.55	0.2270	1.79
	17	9424.25	0.2261	1.84
	18	9929.65	0.2249	1.57
	19	10,433.80	0.2239	1.71
	20	10,935.74	0.2229	1.82
	21	11,434.08	0.2215	1.98
	22	11,926.72	0.2199	2.04
	23	12,413.50	0.2186	1.73
	24	12,897.79	0.2178	1.53
	25	13,380.72	0.2166	1.97
	26	13,858.62	0.2151	1.91
	27	14,332.35	0.2141	1.49
	28	14,804.72	0.2130	1.87
	29	15,272.95	0.2116	1.86
	30	15,737.47	0.2107	1.59
	31	16,199.93	0.2096	1.87
	32	16,658.46	0.2083	1.66
	33	17,114.55	0.2075	1.70
	34	17,568.14	0.2064	1.59
	35	18,019.76	0.2057	1.28
	36	18,471.35	0.2051	1.35
	37	18,922.85	0.2046	1.14
	38	19,375.16	0.2041	1.32
	39	19,826.94	0.2033	1.47
	40	20,277.26	0.2025	1.39
	41	20,726.84	0.2021	0.85
	42	21,178.57	0.2022	0.62
	43	21,633.79	0.2021	1.14
	44	22,089.25	0.2012	1.78
	45	22,540.92	0.1999	1.63
	46	22,989.79	0.1994	0.70

State	$v$	${E}_{v}$ (cm^−1^)	${B}_{v}$ (cm^−1^)	${D}_{v}$×10^7^ (cm^−1^)
(1)^3^Σ^+^	0	278.57	0.2461	1.96
	1	829.37	0.2453	1.73
	2	1393.88	0.2442	1.90
	3	1952.13	0.2430	1.70
	4	2512.64	0.2417	1.87
	5	3067.08	0.2403	1.75
	6	3619.45	0.2391	2.06
	7	4162.04	0.2374	1.77
	8	4701.22	0.2359	1.80
	9	5237.53	0.2351	1.67
	10	5773.28	0.2339	1.88
	11	6303.65	0.2322	1.80
	12	6830.34	0.2313	1.76
	13	7354.17	0.2299	1.79
	14	7874.21	0.2287	1.84
	15	8390.21	0.2275	1.70
	16	8903.40	0.2262	1.79
	17	9412.90	0.2251	1.83
	18	9918.65	0.2239	1.68
	19	10,421.56	0.2226	1.77
	20	10,921.00	0.2214	1.82
	21	11,416.65	0.2202	1.79
	22	11,908.43	0.2189	1.72
	23	12,396.92	0.2178	1.80
	24	12,881.87	0.2166	1.71
	25	13,363.76	0.2154	1.77
	26	13,842.04	0.2142	1.76
	27	14,317.02	0.2131	1.75
	28	14,788.75	0.2119	1.71
	29	15,257.25	0.2107	1.75
	30	15,722.51	0.2097	1.75
	31	16,184.57	0.2085	1.69
	32	16,643.55	0.2074	1.66
	33	17,099.78	0.2064	1.65
	34	17,553.40	0.2054	1.49
	35	18,005.30	0.2046	1.34
	36	18,456.59	0.2039	1.26
	37	18,907.89	0.2034	1.15

State	$v$	${E}_{v}$ (cm^−1^)	${B}_{v}$ (cm^−1^)	${D}_{v}$×10^7^ (cm^−1^)
(2)^3^Δ	0	268.31	0.2437	1.93
	1	815.15	0.2432	1.78
	2	1369.84	0.2416	1.85
	3	1921.58	0.2410	1.82
	4	2471.31	0.2397	1.42
	5	3031.93	0.2395	1.57
	6	3595.88	0.2385	1.96
	7	4150.01	0.2367	2.09
	8	4691.83	0.2348	1.81
	9	5228.57	0.2336	1.81
	10	5761.25	0.2322	1.91
	11	6288.51	0.2310	1.78
	12	6811.99	0.2296	1.74
	13	7332.04	0.2283	1.90
	14	7847.43	0.2272	1.82
	15	8358.92	0.2258	1.66
	16	8867.80	0.2247	1.82
	17	9373.18	0.2237	1.71
	18	9876.10	0.2226	1.55
	19	10,377.73	0.2216	1.78
	20	10,875.92	0.2203	1.93
	21	11,368.69	0.2187	2.03
	22	11,855.10	0.2173	1.78
	23	12,338.18	0.2164	1.54
	24	12,819.98	0.2153	1.84
	25	13,297.47	0.2138	1.97
	26	13,770.00	0.2128	1.54
	27	14,240.95	0.2118	1.77
	28	14,708.29	0.2104	1.86
	29	15,171.56	0.2094	1.54
	30	15,633.00	0.2084	1.80

State	$v$	${E}_{v}$ (cm^−1^)	${B}_{v}$ (cm^−1^)	${D}_{v}$×10^7^ (cm^−1^)
(1)^3^Π	0	291.50	0.2536	2.00
	1	860.03	0.2518	1.83
	2	1437.25	0.2511	1.81
	3	2016.09	0.2494	1.83
	4	2592.79	0.2486	1.78
	5	3168.65	0.2471	1.87
	6	3739.56	0.2459	1.66
	7	4311.43	0.2449	1.83
	8	4878.95	0.2433	2.12
	9	5435.77	0.2415	1.98
	10	5985.38	0.2399	1.64
	11	6535.18	0.2395	1.60
	12	7084.97	0.2379	2.12
	13	7626.12	0.2364	1.72
	14	8165.00	0.2355	1.75
	15	8701.13	0.2340	1.96
	16	9231.76	0.2328	1.70
	17	9759.94	0.2316	1.89
	18	10,283.71	0.2303	1.71
	19	10,804.50	0.2290	1.80
	20	11,321.75	0.2279	1.67
	21	11,836.59	0.2268	1.78
	22	12,348.23	0.2257	1.75
	23	12,856.65	0.2244	2.00
	24	13,359.71	0.2230	1.89
	25	13,858.25	0.2217	1.77
	26	14,353.59	0.2207	1.63
	27	14,846.60	0.2195	1.90
	28	15,335.15	0.2181	1.78
	29	15,820.08	0.2170	1.69
	30	16,302.28	0.2159	1.81
	31	16,780.84	0.2146	1.83
	32	17,255.66	0.2135	1.68
	33	17,727.74	0.2124	1.84
	34	18,196.14	0.2112	1.73
	35	18,661.43	0.2101	1.67
	36	19,124.05	0.2090	1.69
	37	19,584.04	0.2081	1.45
	38	20,042.81	0.2074	1.38
	39	20,500.93	0.2068	1.22
	40	20,959.33	0.2063	1.23

To further verify the truthfulness of our data, we calculated the wavenumbers of the rotational components for P-branch and R-branch for (1)^3^Π_0+_$-$ X^1^Σ_0+_ system as listed in Table 5 by applying the concept of Loomis-Wood diagrams for linear molecules^36^. This method is based on expressing the rovibrational transitions for the P-branch and R-branch as a polynomial of fourth degree in $m$ with $m=- J$ for the P-branch and $m=J+1$ for the R-branch using the following relation:

1 $$ \tilde{v} = \tilde{v}_{0} + (B_{{v^{\prime}}} + B_{{v^{\prime\prime}}} )m + (B_{{v^{\prime}}} - B_{{v^{\prime\prime}}} - D_{{v^{\prime}}} + D_{{v^{\prime\prime}}} )m^{2} - 2\left( {D_{{v^{\prime}}} + D_{{v^{\prime\prime}}} } \right)m^{3} + \left( {D_{{v^{\prime\prime}}} - D_{{v^{\prime}}} } \right)m^{4} $$

where ${\widetilde{v}}_{0}$ is the vibrational transition band center, ${B}_{{v}^{^{\prime}}}$ and ${B}_{{v}^{{^{\prime}}{^{\prime}}}}$ are the rotational constants for the (1)^3^Π_0+_ (upper vibrational state) and X^1^Σ_0+_ (lower vibrational state) respectively, ${D}_{{v}^{^{\prime}}}$ is the centrifugal distortion constant for the upper vibrational state, and ${D}_{{v}^{{^{\prime}}{^{\prime}}}}$ is the centrifugal distortion constant for the lower vibrational state. Comparing these values with those reported by Effantin et al.^19^ yields a good agreement, where the percentage relative difference is 7.2% for the P and R branches. At the same time, the constant shift ($\sim 1162 {\mathrm{cm}}^{-1}$), which corresponds to a relative difference of approximately 7.2% among all presented ro-vibrational energy levels shows that there may have been an experimental setting in^19^ (possible calibration issues) that would have led to a discrepancy in the vibrational transition band center value ${\widetilde{v}}_{0}$, that propagated to all investigated rotational levels.

**Table 5:** The theoretical wavenumbers (in cm^−1^) of the rotation lines of the electronic spectrum of LuF molecule compared with the available experimental values.

(1)^3^Π_0+_$\to$ X^1^Σ_0+_						
$\left({v}^{{^{\prime}}{^{\prime}}}=0-v=0\right)$ Band						
J	P−branch	Shift value (in cm^−1^) $\widetilde{v} {[P\left(J\right)]}_{exp}-\widetilde{v} {[P\left(J\right)]}_{theo}$	Error (%)	R−branch	Shift value (in cm^−1^) $\widetilde{v} {[R\left(J\right)]}_{exp}-\widetilde{v} {[R\left(J\right)]}_{theo}$	Error (%)
0						
1						
2				14,992.29 **16,154.31**^**a**^	1162.02	*7.2*
3				14,992.78 **16,154.8**^**a**^	1162.02	*7.2*
4	14,988.54 **16,150.56**^**a**^	1162.02	*7.2*	14,993.26 **16,155.29**^**a**^	1162.03	*7.2*
5	14,987.96 **16,149.97**^**a**^	1162.01	*7.2*	14,993.72 **16,155.74**^**a**^	1162.02	*7.2*
6	14,987.36 **16,149.41**^**a**^	1162.05	*7.2*	14,994.17 **16,156.24**^**a**^	1162.07	*7.2*
7	14,986.76 **16,148.82**^**a**^	1162.06	*7.2*	14,994.61 **16,156.7**^**a**^	1162.09	*7.2*
8	14,986.14 **16,148.23**^**a**^	1162.09	*7.2*	14,995.04 **16,157.16**^**a**^	1162.12	*7.2*
9	14,985.51 **16,147.63**^**a**^	1162.12	*7.2*	14,995.46 **16,157.62**^**a**^	1162.16	*7.2*
10	14,984.87 **16,147.02**^**a**^	1162.15	*7.2*	14,995.87 **16,158.06**^**a**^	1162.19	*7.2*
11	14,984.22 **16,146.4**^**a**^	1162.18	*7.2*	14,996.26 **16,158.49**^**a**^	1162.23	*7.2*
12	14,983.55 **16,145.78**^**a**^	1162.23	*7.2*	14,996.64 **16,158.91**^**a**^	1162.27	*7.2*
13	14,982.87 **16,145.16**^**a**^	1162.29	*7.2*	14,997.01 **16,159.34**^**a**^	1162.33	*7.2*
14	14,982.18 **16,144.51**^**a**^	1162.33	*7.2*	14,997.37 **16,159.74**^**a**^	1162.37	*7.2*
15	14,981.48 **16,143.85**^**a**^	1162.37	*7.2*	14,997.72 **16,160.15**^**a**^	1162.43	*7.2*
16	14,980.77 **16,143.18**^**a**^	1162.41	*7.2*	14,998.05 **16,160.53**^**a**^	1162.48	*7.2*
17	14,980.05 **16,142.56**^**a**^	1162.51	*7.2*	14,998.37 **16,160.93**^**a**^	1162.56	*7.2*
18	14,979.31 **16,141.86**^**a**^	1162.55	*7.2*	14,998.68 **16,161.3**^**a**^	1162.62	*7.2*
19	14,978.56 **16,141.18**^**a**^	1162.62	*7.2*	14,998.98 **16,161.66**^**a**^	1162.68	*7.2*
20	14,977.81 **16,140.56**^**a**^	1162.75	*7.2*	14,999.27 **16,162.03**^**a**^	1162.76	*7.2*

Percentage relative error values are in [italics].

^a^Ref.^19^. Experimental values are indicated in bold.

**Figure 4:**
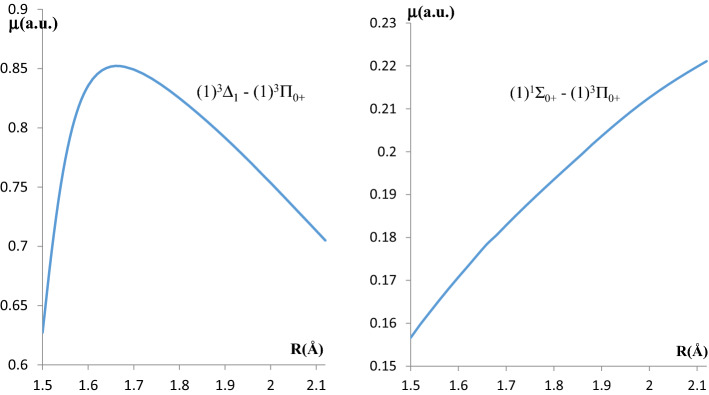
The transition dipole moment curves for the X^1^Σ_0+_−(1)^3^Π_0+_ and (1)^3^Δ_1_ − (1)^3^Π_0+_ transitions of LuF molecule.

The fine structure selection rules state that the transitions Σ−Δ, ΔΩ > 1, and 0^+^−0^−^ are forbidden^20^. Consequently, we analyzed only the transitions ^1^Π − ^1^Σ^+^, ^1^Π − ^1^Δ among the lowest states shown in Fig. 3. More precisely, we present their Transition Dipole Moment (TDM) curves in the considered region $1.5~{\text{{\AA}}} \le R \le 2.12~{\text{{\AA}}}$ in Fig. 4. We then deduced the electronic emission coefficients proposed by Hilborn et al.^37^, based on the values of the transitions’ dipole moments at the equilibrium positions of the upper states for each electronic transition $\left|{\mu }_{21}\right|$:

2 $$ {\upomega }_{{{\text{ij}}}} = 2{{ \pi \nu }}_{{{\text{ij}}}} $$

3 $$ {\text{A}}_{{{\text{ij}}}} = \frac{{2{\upomega }_{{{\text{ij}}}}^{3} {\upmu }_{{{\text{ij}}}}^{2} }}{{3{\upvarepsilon }_{0} {\text{hc}}^{3} }} $$

4 $$ {\upgamma }_{{{\text{cl}}}} = \frac{{{\text{e}}^{2} {\upomega }_{{{\text{ij}}}}^{2} }}{{6{{ \pi \varepsilon }}_{0} {\text{m}}_{{\text{e}}} {\text{c}}^{3} }} $$

5 $$ {\text{f}}_{{{\text{ij}}}} = \frac{{ - {\text{ A}}_{{{\text{ij}}}} }}{{3{{ \gamma }}_{{{\text{cl}}}} }} $$

${\omega }_{21}$ is the emission angular frequency and ${A}_{21}$ is the Einstein coefficients for spontaneous emissions. For the perpendicular transitions with ΔΛ =  ± 1 (or ΔΩ =  ± 1) such as ^1^Π − ^1^Σ^+^, ^1^Π − ^1^Δ, the Einstein coefficient A_ij_ must be divided by an additional factor of two^38^ depending on the exact definition of ${\mu }_{ij}$. The constants ${\upvarepsilon }_{0}$ and m_e_ are respectively the vacuum permittivity and the mass of the electron. $\left|{f}_{21}\right|, {\gamma }_{cl}, \mathrm{and} {\upnu }_{\mathrm{ij}}$ are respectively the oscillator strength constant, the classical radiative decay rate of the single-electron oscillator, and the transition frequency between the two states. *h* and *c* represent the Planck constant and the speed of light, respectively. The calculated values of these constants for the two transitions (1)^3^Π_0+_ −X^3^Σ_0+_ and (1)^3^Π_0+_−(1)^3^Δ_1_ are given in Table 6. No comparison of these results with literature is available since they are given here for the first time. However, the value of the radiative lifetime τ will be discussed in the next section.

**Table 6:** The transition dipole moment values at the upper state equilibrium position $\left|{\mu }_{21}\right|$, the emission angular frequency ${\omega }_{21}$, the Einstein spontaneous coefficients ${A}_{21}$, the spontaneous radiative lifetime ${\tau }_{21}$, the classical radiative decay rate of the single-electron oscillator ${\gamma }_{cl}$, and the emission oscillator strength ${f}_{21}$ of some transitions among the doublet states of LuF molecule.

LuF-SO						
Transition	$\left|{\upmu }_{21}\right|$($\mathrm{a}.\mathrm{u}.$)	${\upomega }_{21} \times {10}^{-15}$ ($\mathrm{rad }{\mathrm{s}}^{-1}$)	${\mathrm{A}}_{21}$ (${\mathrm{s}}^{-1}$)	${\uptau }_{21}$($\mathrm{s}$)	${\upgamma }_{\mathrm{cl}}\times {10}^{-6}$(${\mathrm{s}}^{-1}$)	$\left|{\mathrm{f}}_{21}\right|$
X^1^Σ_0+_−(1)^3^Π_0+_	0.2126	2.826	3.1 × 10^5^	3.235 × 10^–6^	50.0	0.00206
(1)^3^Δ_1_−(1)^3^Π_0+_	0.7536	0.2807	3.8 × 10^3^	2.625 × 10^–4^	0.494	0.00258

## Laser cooling study of LuF molecule

The difference in the values of the equilibrium positions ΔR_e_ between the ground X^1^Σ_0+_ and (1)^3^Π_0+_ and (1)^3^Δ_1_ states of LuF molecule is minimal, which is an encouraging factor in verifying the laser cooling feasibility for this molecule. The main criteria to keep a closed-loop cycle in a laser cooling process are:

1. A highly diagonal Franck–Condon factor (FCF) among the lowest vibrational levels of the ground and considered excited states.
2. The absence of an intervening intermediate state, unless it was found possible to include it within the laser cooling scheme.
3. A short radiative lifetime of a given transition (in the range of ns to μs) ensures a rapid spontaneous deexcitation of the molecules to provide a high number of cycles per second.

**Figure 5:**
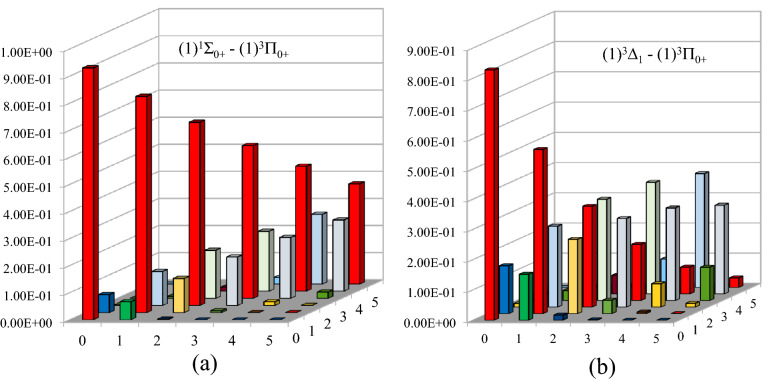
The Franck-Condon factor for X^1^Σ_0+_ − (1)^3^Π_0+_ and (1)^3^Δ_1_ − (1)^3^Π_0+_ transitions of LuF molecule.

We consequently calculated the FCF values among specific states using LEVEL 11 program^39^. Our results for the allowed transitions (1)^3^Π_0+_ −X^3^Σ_0+_ and (1)^3^Π_0+_ − (1)^3^Δ_1_ show a diagonal FCF among the first three vibrational levels for the two transitions, as shown in Fig. 5.

To probe how substantially an intermediate state influences a given cooling cycle, one can rely upon the vibrational branching ratio loss $\left( {\gamma = \left( {{\text{A}}_{{\nu^{\prime\prime}\nu^{\prime} - {\text{ Excited}}/{\text{Intermediate}}}} } \right)/({\text{A}}_{{\nu^{\prime\prime}\nu - {\text{ Excited}}/{\text{Ground}}}} )} \right)$ between the considered intermediate state and the excited state involved in the cooling process. Here, A_ν′′ν′−Excited/Intermediate)_ is the Einstein coefficient for transitions between the excited and Intermediate states, and A_ν′′ν−excited/ground_ is that for transitions between the excited and ground-state. If the value of γ is less than 10^–4^, then the intermediate state should have a minimal effect on the cooling cycle^40^.

In our case, we follow a similar procedure to understand the implication of the intermediate state (1)^3^Δ_1_, in a cooling loop cycle consisting of the ground state X^1^Σ_0+_, and the excited state (1)^3^Π_0+_. In general, Einstein's coefficient ${A}_{\nu {^{\prime}}\nu }$ among vibrational levels can be written as the following^41^:

6 $$ A_{{\nu^{\prime}\nu }} = (\left( {3.1361891} \right)\left( {10^{ - 7} } \right)(\Delta E)^{3} \left( {\left. {\psi_{{\nu^{\prime}}} } \right|M\left( r \right)\left| {\psi_{\nu } } \right.)^{2} } \right) $$

where ΔE is the emission frequency (in cm^−1^), and M(r) is the electronic transition dipole moment between the two electronic states that are considered (in Debye).

Our calculated value of the transition dipole moment, obtained with the Molpro software^30^, is vertical (given as μ_x_, μ_y_, and μ_z_)). In our calculations, we choose the highest value of these transition matrix elements (μ_x_ in this case_)_. Consequently, we considered the Einstein coefficient ${A}_{\nu {^{\prime}}\nu }$ (Eq. (2)) to be:

7 $$ A_{{\nu^{\prime}\nu }} = (\left( {3.1361891} \right)\left( {10^{ - 7} } \right)(\Delta E)^{3} \left( {\left. {\psi_{{\nu^{\prime}}} } \right|\mu_{x} \left| {\psi_{\nu } } \right.)^{2} } \right) $$

The values of the vibrational branching ratio for the first five vibrational levels, which represents the percentage of transition probability between two vibrational levels, are given in Tables 7, 8 and are obtained by using the formula^42^:

8 $$ R_{{\nu^{\prime}\nu }} = \frac{{A_{{\nu^{\prime}\nu }} { }}}{{\mathop \sum \nolimits_{\nu } A_{{\nu^{\prime}\nu }} }} $$

**Table 7:** The radiative lifetimes τ, and the vibrational branching ratio of the vibrational transitions between the electronic states (1)^3^Π_0+_−X^1^Σ_0+_ of the molecule LuF.

LuF-SO								
	ν_′′_((1)^3^Π_0+_) = 0		1	2	3	4	5	6
ν (X^1^Σ_0+_) = 0	A_ν′′ν_	269,977.6372	18,918.19075	642.7389436	10.37449812	0.008082286	0.007478636	1.08E−04
	R_ν′′ν_	0.93012820	0.06540194	0.00224561	0.00003631	0.00000003	0.00000003	0.00000000
ν = 1	A_ν′′ν_	19,607.00055	231,378.8478	35,777.64278	1968.533459	47.14847216	0.155388532	0.027200272
	R_ν′′ν_	0.06755013	0.79989809	0.12500066	0.00689015	0.00016637	0.00000057	0.00000016
ν = 2	A_ν′′ν_	662.5329264	36,962.71764	193,724.8751	50,607.33398	3957.673996	133.1030565	0.860364798
	R_ν′′ν_	0.00228256	0.12778354	0.67683995	0.17713293	0.01396533	0.00049230	0.00000496
ν = 3	A_ν′′ν_	11.23597355	1953.886076	52,100.03828	161,755.6346	63,227.53364	6604.88765	293.2955742
	R_ν′′ν_	0.00003871	0.00675476	0.18202819	0.56616793	0.22310912	0.02442886	0.00169205
ν = 4	A_ν′′ν_	0.114685072	46.11648268	3853.65538	64,779.67216	131,027.7702	73,541.16762	9890.39006
	R_ν′′ν_	0.00000040	0.00015943	0.01346398	0.22673815	0.46235381	0.27199960	0.05705855
ν = 5	A_ν′′ν_	0.000877974	0.642376596	118.627261	6336.161712	75,768.21386	105,399.3421	81,877.02166
	R_ν′′ν_	0.00000000	0.00000222	0.00041446	0.02217747	0.26736105	0.38983035	0.47235587
ν = 6	A_ν′′ν_	7.34E−05	0.007381814	2.04282127	244.8646484	9364.514784	84,693.67356	81,275.97982
	R_ν′′ν_	0.00000000	0.00000003	0.00000714	0.00085706	0.03304429	0.31324830	0.46888841
Sum (s^−1^) = A_ν′′ν_		290,258.5223	289,260.4085	286,219.6205	285,702.5751	283,392.8630	270,372.3369	173,337.5748
τ:(s) = 1/A_ν′′ν_		3.4452E−06	3.45709E−06	3.49382E−06	3.50014E−06	3.52867E−06	3.6986E−06	5.76909E−06
τ:(μs)		3.45	3.46	3.49	3.50	3.53	3.70	5.77

**Table 8:** The radiative lifetimes τ, and the vibrational branching ratio of the vibrational transitions between the electronic states (1)^3^Π_0+_−(1)^3^Δ_1_ of the molecule LuF.

LuF-SO								
	ν′′((1)^3^Π_0+_) = 0		1	2	3	4	5	6
v′ ((1)^3^Δ_1_) = 0	A_ν_′′_ν_′	3795.717704	420.4072658	420.4072658	0.014461864	7.04748E−05	0.000178738	9.99366E−05
	R_ν_′′_ν_′	0.98478369	0.05448771	0.05448771	0.00000185	0.00000001	0.00000002	0.00000001
v′ = 1	A_ν_′′_ν_′	58.52845986	7182.184544	7182.184544	0.155152468	0.071005714	0.00084819	0.00012794
	R_ν_′′_ν_′	0.01518497	0.93086119	0.93086119	0.00001986	0.00000904	0.00000011	0.00000002
v′ = 2	A_ν_′′_ν_′	0.12764095	112.6588205	112.6588205	1185.079902	0.233541534	0.185820348	0.004076242
	R_ν_′′_ν_′	0.00003312	0.01460137	0.01460137	0.15171184	0.00002974	0.00002355	0.00000054
v′ = 3	A_ν_′′_ν_′	−0.00141388	0.410281126	0.410281126	6413.38877	1538.539034	0.291628088	0.393386436
	R_ν_′′_ν_′	−0.00000037	0.00005318	0.00005318	0.82103072	0.19594947	0.00003696	0.00005190
v′ = 4	A_ν_′′_ν_′	−0.003770594	−0.004047702	−0.004047702	211.2423738	6056.0312	1872.106295	0.309794184
	R_ν_′′_ν_′	−0.00000098	−0.00000052	−0.00000052	0.02704288	0.77130060	0.23729473	0.00004087
v′ = 5	A_ν_′′_ν_′	−0.00134734	−0.016112822	−0.016112822	1.516054618	254.470936	5722.200736	2167.619392
	R_ν_′′_ν_′	−0.00000035	−0.00000209	−0.00000209	0.00019408	0.03240961	0.72530501	0.28597214
v′ = 6	A_ν_′′_ν_′	−0.000308208	−0.006421308	−0.006421308	−0.009631366	2.367540836	294.5860572	5411.500234
	R_ν_′′_ν_′	−0.00000008	−0.00000083	−0.00000083	−0.00000123	0.00030153	0.03733961	0.71393452
Sum (s^−1^) = A_ν_′′_ν_′		3854.366965	7715.63433	7715.63433	7811.387084	7851.713329	7889.371564	7579.827111
τ: (s) = 1/A_ν_′′_ν_′		0.000259446	0.000129607	0.000129607	0.000128018	0.000127361	0.000126753	0.000131929
τ: (ms)		0.259	0.130	0.130	0.128	0.127	0.127	0.132

Finally, each transition's radiative lifetimes are calculated using τ(s) = $1/{\sum }_{\nu }{A}_{\nu {^{\prime}}\nu }$_,_ and presented in Table 7 and Table 8. Up to our knowledge, the radiative lifetimes of LuF molecule spin–orbit states are presented here for the first time in the literature. One can notice that the spontaneous emission of the transition (1)^3^Π_0+_ − X^3^Σ_0+_ is dominant over that of (1)^3^Π_0+_ − (1)^3^Δ_1_, where the FCF and the radiative lifetime of the former are f_00_ = 0.930636 and τ = 3.45 μs while those of the later are f_00_ = 0.828539 and τ = 0.259 ms. The variation in the radiative lifetime for the two transitions is due to the difference in energy ΔE, which is much more important between the ground state X^1^Σ_0+_ and the excited state (1)^3^Π_0+_, compared to that between the excited state (1)^3^Π_0+_ and the intermediate state (1)^3^Δ_1._ The comparison of these values for the radiative lifetime with those calculated by using Hilborn emission coefficients^37^ that are given in Table 6 for the two transitions (1)^3^Π_0+_ − X^3^Σ_0+_ and (1)^3^Π_0+_ − (1)^3^Δ_1_ shows an excellent agreement with the relative differences 6.2% and 1.3% respectively.

Our calculated value for the vibrational branching loss ratio γ  = γ_Δ_/γ_Σ_ = 0.02812 where, γ_Δ_ and γ_Σ_ represent the total emission rate of the (1)^3^Π_0+_ − (1)^3^Δ_1_, and (1)^3^Π_0+_ − X^1^Σ_0+_ transitions, respectively. The order of this ratio is two times higher than the minimum required value of 10^–4^^40^. Consequently, the intermediate state (1)^3^Δ_1_ must be considered while setting a convenient laser cooling scheme. At the same time, the forbidden transitions (1)^3^Π_0+_ − (1)^3^Π_0−_, (1)^3^Π_0+_ − (1)^3^Δ_2_, and X^1^Σ_0+_ − (1)^3^Δ_2_^20^ do not disturb the transition X^1^Σ_0+_ − (1)^3^Π_0+_.

Laser cooling schemes with an intermediate state have already been proposed in the literature^43,44^. We use the technique proposed by Yuan et al.^41^ to include the intermediate state in the laser cooling cycle. To this end, one must calculate the Einstein coefficients for transitions among the three involved electronic states, i.e., X^1^Σ_0+_, (1)^3^Π_0,_ and (1)^3^Δ_1._ For the two transitions (1)^3^Π_0+_ − X^3^Σ_0+_ and (1)^3^Π_0+_ − (1)^3^Δ_1_, the values of the vibrational branching ratio for the first five vibrational levels are given in Table 9 by using the formulas:

9 $$  \left( {\text{1}} \right)^{{\text{3}}} \Pi _{{0 + }}  - {\text{X}}^{{\text{1}}} \Sigma _{{0 + }}  \to R_{{\nu ^{\prime\prime}\nu }} ~ = \frac{{A_{{\nu ^{\prime\prime}\nu }} {\text{~}}}}{{\mathop \sum \nolimits_{\nu } A_{{\nu ^{\prime\prime}\nu }}  + \mathop \sum \nolimits_{{\nu ^{\prime}}} A_{{\nu ^{\prime\prime}\nu ^{\prime}}} }}  $$

10 $$ \left( {1} \right)^{{3}} \Pi_{0 + } - \left( {1} \right)^{{3}} \Sigma_{{1}} \to R^{\prime}_{{\nu^{\prime\prime}v^{\prime}}} = \frac{{A_{{\nu^{\prime\prime}v^{\prime}}} }}{{\mathop \sum \nolimits_{\nu } A_{{\nu^{\prime\prime}\nu }} + \mathop \sum \nolimits_{{\nu^{\prime}}} A_{{\nu^{\prime\prime}\nu^{\prime}}} }} $$

**Table 9:** The radiative lifetimes τ, and the vibrational branching ratio of the vibrational transitions between the electronic states (1)^3^Π_0+_−X^1^Σ_0+_ and (1)^3^Π_0+_−(1)^3^Δ_1_ of the molecule LuF.

	ν′′((1)^3^Π_0+_) = 0		1	2	3	4	5
ν (X^1^Σ_0+_) = 0	A_ν_′′_ν_	269,977.6372	18,918.19075	642.7389436	10.37449812	0.008082286	0.007478636
	R_ν_′′_ν_	0.917938781	0.063702883	0.002187566	0.002187566	3.9262E−08	9.10342E−08
ν = 1	A_ν_′′_ν_	19,607.00055	231,378.8478	35,777.64278	1968.533459	47.14847216	0.155388532
	R_ν_′′_ν_	0.066664878	0.77911783	0.121769445	0.006860641	0.000229037	1.89148E−06
ν = 2	A_ν_′′_ν_	662.5329264	36,962.71764	193,724.8751	50,607.33398	3957.673996	133.1030565
	R_ν_′′_ν_	0.002252648	0.124463894	0.659343902	0.176374314	0.019225541	0.001620207
ν = 3	A_ν_′′_ν_	11.23597355	1953.886076	52,100.03828	161,755.6346	63,227.53364	6604.88765
	R_ν_′′_ν_	3.82029E−05	0.006579285	0.177322827	0.563743174	0.307145956	0.080398471
ν = 4	A_ν_′′_ν_	0.114685072	46.11648268	3853.65538	64,779.67216	131,027.7702	73,541.16762
	R_ν_′′_ν_	3.89936E−07	0.000155287	0.013115942	0.225767084	0.636505134	0.895185167
ν′ ((1)^3^Δ_1_) = 0	A_ν_′′_ν_′	3795.717704	420.4072658	420.4072658	0.014461864	7.04748E−05	0.000178738
	R′_ν_′′_ν_′	0.012905648	0.00141563	0.001430859	5.04018E−08	3.42352E−10	2.17571E−09
ν′ = 1	A_ν_′′_ν_′	58.52845986	7182.184544	7182.184544	0.155152468	0.071005714	0.00084819
	R′_ν_′′_ν_′	0.000199	0.024184441	0.024444613	5.4073E−07	3.44931E−07	1.03246E−08
ν′ = 2	A_ν”ν’_	0.12764095	112.6588205	112.6588205	1185.079902	0.233541534	0.185820348
	R′_ν_′′_ν_′	4.33986E−07	0.000379354	0.000383435	0.004130185	1.1345E−06	2.26191E−06
ν′ = 3	A_ν_′′_ν_′	0.00141388	0.410281126	0.410281126	6413.38877	1538.539034	0.291628088
	R′_ν_′′_ν_′	4.80727E−09	1.38153E−06	1.39639E−06	0.022351643	0.007473897	3.54986E−06
ν′ = 4	A_ν_′′_ν_′	0.003770594	0.004047702	0.004047702	211.2423738	6056.0312	1872.106295
	R′_ν_′′_ν_′	1.28202E−08	1.36298E−08	1.37764E−08	0.000736212	0.029418916	0.022788349
$\mathop \sum \limits_{\nu } A_{{\nu^{\prime\prime}\nu }} + \mathop \sum \limits_{{\nu^{\prime}}} A_{{\nu^{\prime\prime}\nu^{\prime}}}$		294,112.9003	296,975.4237	293,814.6154	286,931.4294	205,855.0092	82,151.90596
τ = 1/($\mathop \sum \limits_{\nu } A_{{\nu^{\prime\prime}\nu }} + \mathop \sum \limits_{\nu ^{\prime}} A_{{\nu^{\prime\prime}\nu^{\prime}}}$		3.40005E−06	3.36728E−06	3.40351E−06	3.48515E−06	4.85779E−06	1.21726E−05
τ (μs)		3.40	3.37	3.40	3.49	4.86	12.17

${A}_{\nu {^{\prime}}{^{\prime}}\nu }$ and ${A}_{v{^{\prime}}{^{\prime}}v{^{\prime}}}$ are the Einstein coefficients for the transitions (1)^3^Π_0+_−X^1^Σ_0+_ and (1)^3^Π_0+_−(1)^3^Δ_1_, respectively. For the main optical cycle of the transition (1)^3^Π_0+_ − X^1^Σ_0+_, the number of cycles (N) for photon absorption/emission among vibrational levels (denoted as a, b, c, etc.…) is reciprocal to the total loss:

11 $$ N = \frac{1}{{1 - \left[ {(R_{00} + R^{\prime}_{00} + R_{0a} + R^{\prime}_{0a } \ldots ) + (R_{0b} + R^{\prime}_{0b} + R_{0c} + R^{\prime}_{0c} \ldots .)\left( {R_{a0} + R^{\prime}_{a0} + R_{aa} + R^{\prime}_{ab} + \ldots .} \right)} \right]}} $$

Having the value of N, the experimental parameters needed to realize the cooling of a molecule can be obtained. If *k*_*b*_ and *h* are the Boltzmann and Planck constants, and m is the mass of the molecule, the mathematical expressions of these parameters are^45^:

12 $$ V = \frac{hN}{{m\lambda_{00} }} $$

13 $$ {\text{T}}_{{{\text{ini}}}} = \frac{{mV^{2} }}{{2k_{B} }} $$

14 $$ {\text{a}}_{{{\text{max}}}} = \frac{{hN_{e} }}{{N_{tot} m\lambda_{00} \tau }} $$

15 $$ L = \frac{{k_{B} T_{ini} }}{{ma_{max} }} $$

where V and T_ini_ are the initial velocity and temperature of the molecule, respectively. The maximum acceleration is a_max,_ and the slowing distance is L. N_e_ is the number of excited states in the main cycling transition and N_tot_ is the number of the excited states connected to the ground state plus N_e_.

**Figure 6:**
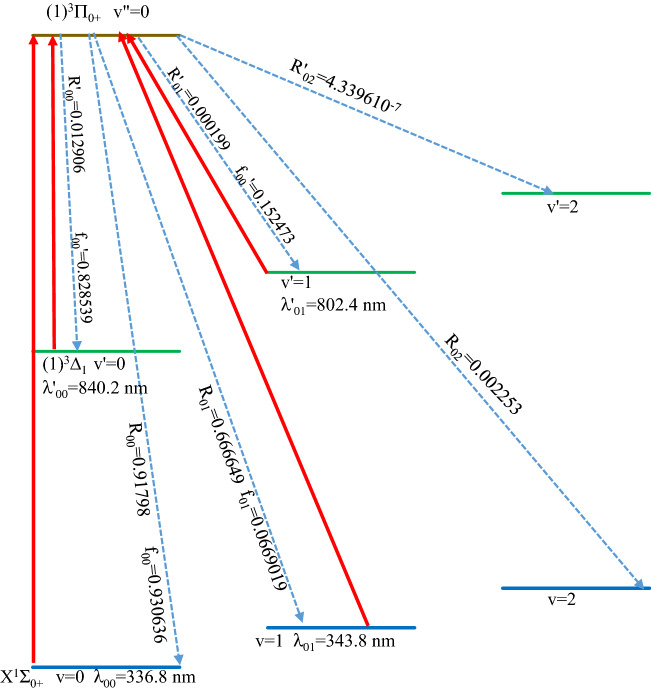
The laser cooling scheme of X^1^Σ_0+_ − (1)^3^Π_0+_ and (1)^3^Δ_1_ − (1)^3^Π_0+_ transitions of LuF molecule.

We considered the cooling scheme presented in Fig. 6 to obtain suitable experimental values. The driving lasers are given in solid lines for the two transitions along with their corresponding wavelengths. Dotted lines represent the spontaneous decays with the values of their FCF (f_ν′′ν_ and f′_ν′′ν′_) and the vibrational branching ratios *R*_ν′′ν_ and *R*’_ν′′ν′_. The suggested scheme includes the two transitions X^1^Σ_0+_ − (1)^3^Π_0+_ and (1)^3^Π_0+_ − (1)^3^Δ_1_. The wavelength of the main cycling laser for the transition X^1^Σ_0+_ − (1)^3^Π_0+_ is λ_00_ = 336.8 nm, while that of the repump laser is λ_01_ = 343.8 nm. Since the influence of the intermediate state (1)^3^Δ_1_ cannot be ignored, there is a need for two additional lasers to handle the loss to the vibrational levels for the transition (1)^3^Π_0+_ − (1)^3^Δ_1_, at λ′_00_ = 840.2 nm and λ′_01_ = 802.4 nm.

The value of N for this scheme is calculated as the following:

16 $$ {\text{N}} = \frac{1}{{1 - [\left( {R_{00} + R^{\prime}_{00} + R_{01} + R^{\prime}_{01 } } \right)}} $$

The corresponding experimental parameters for this scheme are N = 442, L = 1.04 cm,V = 2.73 m/s, T_ini_ = 86.7 mK, a_max_ = 358 m/s^2^, and N_e_/N_tot_ = 1/3. The temperatures that can be reached during the cooling process can be obtained by calculating the Doppler limit temperature T_D_ and the recoil temperature T_r_^42^:

$${T}_{D}=\frac{h}{4{k}_{B}\pi \tau }=1108.6 nK$$ and $${T}_{r}=\frac{{h}^{2}}{m{k}_{B}{\uplambda }_{00}^{2}}=888 mK$$

Suppose T_i_ is the temperature of the LuF molecules obtained by using laser ablation to produce the atoms of the LuF molecule^46^, typically in the order of T_i_ = 7000 K. In that case, there is a need for an intermediate process for the molecules to reach the mK regime. This regime can be obtained by collisions between LuF hot molecules of mass M, and cold buffer helium gas of mass m and temperature T_B_. After N collision, the temperature T_N_ of the molecule is given by^47^:

17 $$ T_{N} = \, T_{B} + \, \left( {T_{i} - \, T_{B} } \right){\text{exp}}\left( { - 2Mm/\left( {M \, + \, m} \right)^{2} } \right)N $$

We suggest a pre-cooling temperature for LuF molecule T_N_ = 86.7 mK, corresponding to T_ini_ of the laser cooling process, and a helium gas temperature T_B_ = 2 K. From Eq. (6), the number of collisions in the buffer cell equals N = 285. At low temperatures in the buffer gas cell, the collision between the molecules can be ignored. If the density of helium n_He_ = 5 × 10^14^ cm^3^ and the collision cross-section σ_X−He_ = 10^–14^ cm^2^ the average distance (mean free path) λ between two collisions is given by^48^

18 $$ \lambda \, = { 1}/\left( {\left( {\sigma_{{{\text{X}} - {\text{He}}}} \cdot {\text{ n}}_{{{\text{He}}}} } \right)\left( {{1} + {\text{M}}/{\text{m}}} \right)^{{{1}/{2}}} } \right) $$

The corresponding value of λ = 0.0287 cm. Based on the rules of the kinetic theory of ideal gases, the molecules in the buffer gas cell will be thermalized during the time^48^:

19 $$ t = \mathop \sum \limits_{N = 0}^{98} \frac{\lambda }{{\sqrt {\frac{{3k_{b} }}{M}\left( {T_{i} - T_{B} } \right)e^{ - N/\kappa } + T_{i} } }} $$

where $\kappa =\frac{{(M+m)}^{2}}{2mM}$. During this short time, t = 0.444 ms in the buffer gas, the LuF molecules will reach a suitable before being sent to the Doppler laser cooling setup.

## Conclusion

The adiabatic potential energy curves for the singlet and triplet electronic states of the LuF molecule have been investigated with spin–orbit calculation upon employing the MRCI + Q technique with Davidson correction. The calculation of the spectroscopic constants and the FCF show the candidacy of the LuF molecule for a direct laser cooling between the two states X^1^Σ_0+_ and (1)^3^Π_0+_ with the intermediate state (1)^3^Δ_1_. Since the influence of this state cannot be ignored, the study of the laser cooling of this molecule has been done by taking into consideration the two transitions (1)^3^Δ_1_−(1)^3^Π_0+_ and X^1^Σ_0+_ − (1)^3^Π_0+_. Correspondingly, a total branching ratio is investigated with a short radiative time (τ = 3.40 μs) along with the slowing distance, the number of cycles (N) for photon absorption/emission, and the Doppler and recoil temperatures. The time needed to thermalize the molecules in the buffer gas cell is calculated, with the number of collisions in this cell between the molecules with the helium atoms and the mean free path between two collisions. This study of the laser cooling of the molecule LuF paves the way to an experimental laser cooling of this molecule.

## Data Availability

All data generated or analysed during this study are included in this published article.
